# Cross-Sensory EEG Emotion Recognition with Filter Bank Riemannian Feature and Adversarial Domain Adaptation

**DOI:** 10.3390/brainsci13091326

**Published:** 2023-09-14

**Authors:** Chenguang Gao, Hirotaka Uchitomi, Yoshihiro Miyake

**Affiliations:** Department of Computer Science, Tokyo Institute of Technology, Yokohama 226-8502, Japan; uchitomi@c.titech.ac.jp (H.U.); miyake@c.titech.ac.jp (Y.M.)

**Keywords:** EEG emotion recognition, cross-sensory emotion recognition, Riemannian feature extraction, adversarial domain adaptation

## Abstract

Emotion recognition is crucial in understanding human affective states with various applications. Electroencephalography (EEG)—a non-invasive neuroimaging technique that captures brain activity—has gained attention in emotion recognition. However, existing EEG-based emotion recognition systems are limited to specific sensory modalities, hindering their applicability. Our study innovates EEG emotion recognition, offering a comprehensive framework for overcoming sensory-focused limits and cross-sensory challenges. We collected cross-sensory emotion EEG data using multimodal emotion simulations (three sensory modalities: audio/visual/audio-visual with two emotion states: pleasure or unpleasure). The proposed framework—filter bank adversarial domain adaptation Riemann method (FBADR)—leverages filter bank techniques and Riemannian tangent space methods for feature extraction from cross-sensory EEG data. Compared with Riemannian methods, filter bank and adversarial domain adaptation could improve average accuracy by 13.68% and 8.36%, respectively. Comparative analysis of classification results proved that the proposed FBADR framework achieved a state-of-the-art cross-sensory emotion recognition performance and reached an average accuracy of 89.01% ± 5.06%. Moreover, the robustness of the proposed methods could ensure high cross-sensory recognition performance under a signal-to-noise ratio (SNR) ≥ 1 dB. Overall, our study contributes to the EEG-based emotion recognition field by providing a comprehensive framework that overcomes limitations of sensory-oriented approaches and successfully tackles the difficulties of cross-sensory situations.

## 1. Introduction

Emotion recognition is identifying and interpreting human emotions based on cues such as facial expressions, vocal intonations, physiological signals, and behavioral patterns [1]. It involves using algorithms and machine learning to analyze these cues and classify them into different emotional states, such as happiness, sadness, anger, or fear. Emotion recognition is applicable in various fields, including psychology, human–computer interaction, and healthcare [2]. Electroencephalography (EEG) is a neuroimaging technique that measures the electrical activity in the brain using electrodes placed on the scalp. It records collective electrical signals generated by neuron firings in the brain. EEG provides valuable insights into brain activity and is widely used in neuroscience research, clinical diagnostics, and brain–computer interface (BCI) systems [3]. EEG signals can be analyzed to detect patterns associated with specific mental states, cognitive processes, and emotional responses. EEG, owing to its non-invasiveness and high sensitivity to various emotions, has recently gained increasing attention as a physiological signal for emotion recognition [4]. The affective brain–computer interface (aBCI) combines the principles of EEG and emotion recognition to create a direct communication pathway between the brain and an external device or computer system. This allows individuals to control external devices or interact with computer systems using their emotional states as inputs. The aBCI systems use EEG signals to detect and interpret emotional states, translated into commands or actions for connected devices [5].

The current trend in EEG-based aBCI focuses on improving the accuracy and reliability of emotion recognition using EEG signals [6]. The general concept of EEG-aBCI comprises data acquisition, preprocessing, feature extraction, classification/regression, model evaluation, and emotion recognition. The mean-focused parts are feature extraction and classification/regression. Recently, various machine learning-inspired methods have been applied in EEG emotion recognition. Liu et al. [7] proposed an EEG emotion recognition model that combines an attention mechanism and a pre-trained convolution capsule network to effectively recognize emotions, enhancing emotion-related information in EEG signals. Li et al. [8] presented the Frontal Lobe Double Dueling Deep Q Network (FLD3QN), a model inspired by the Papez circuit theory and reinforcement learning neuroscience, utilizing EEG signals from the frontal lobe to enhance emotion perception. Padhmashree and Bhattacharyya [9] presented a novel four-stage method for human emotion recognition using multivariate EEG signals, achieving exceptional performance by incorporating deep residual networks and time-frequency-based analysis. Wei et al. [10] proposed that the Transformer Capsule Network (TC-Net) achieves state-of-the-art EEG-based emotion recognition performance on DEAP and DREAMER datasets. This demonstrates its effectiveness in capturing global contextual information through an EEG Transformer and Emotion Capsule modules. Cui et al. [11] integrated signal complexity, spatial brain structure, and temporal context through 4D feature tensors, Convolutional Neural Networks, and Bidirectional Long-Short Term Memory, achieving high accuracy (94% for DEAP and 94.82% for SEED datasets) by deep decoding EEG signals and extracting key emotional features. Algarni et al. [12] developed a deep learning-based approach for EEG-based emotion recognition, contributing to improved accuracy in diagnosing psychological disorders by achieving high accuracies (99.45% valence, 96.87% arousal, 99.68% liking) through a multi-phase process involving data selection, feature extraction, selection, and classification using a stacked bi-directional Long Short-Term Memory (Bi-LSTM) Model. To enhance human–computer interaction, Islam et al. [13] proposed PCC-CNN, which is a deep machine-learning-based Convolutional Neural Network (CNN) model for emotion recognition using EEG signals in which Pearson’s correlation coefficient-featured images are employed for channel correlation analysis in sub-bands. Peng et al. [14] introduced a unified framework, GFIL (graph-regularized least square regression with feature importance learning), for EEG-based emotion recognition and highlighted the significance of the Gamma band and prefrontal/central region channels in emotion recognition. Huang et al. [15] presented an EEG-based emotion detection system that utilized short EEG segments of 1 s, incorporating a novel feature extraction algorithm—asymmetric spatial filtering—into a filter bank framework. Chen et al. [16] proposed an emotion recognition method using EEG signals, which involved extracting the energy means of detail coefficients as feature values and using a support vector machine (SVM) for classification. They demonstrated the validity of the feature values and provided a theoretical basis for implementing effective human–computer interaction. Wu et al. [17] highlighted the importance of Riemannian feature extraction in EEG-based emotion recognition, demonstrating that the proposed independent component analysis with a Riemannian manifold and long short-term memory networks (ICRM-LSTM) model outperformed existing methods by effectively addressing the uncertain ordering in independent component analysis (ICA). Wang et al. [18] proposed a domain-adaptation symmetric positive definite (SPD) matrix network (daSPDnet) that effectively captured shared emotional representations among different individuals using Riemannian feature extraction. Based on the aforementioned EEG emotion recognition studies, the filter bank was highly robust, and Riemannian feature extraction is an emerging field for effective feature extraction.

Recent research has revealed that multimodal stimulation with cross-sensory emotions is crucial in human–computer interaction and affective computing (AC). Ranasinghe et al. [19] demonstrated that wearable accessories such as head-mounted displays (HMD), with wind and thermal stimuli, significantly improve sensory and realism factors, enhancing the sense of presence compared to traditional virtual reality (VR) experiences. Zhu et al. [20] explored the emerging field of multimodal sentiment analysis, which integrates text, visual, and audio information to infer sentiment polarity, aiming to provide researchers with insights and inspiration for developing effective models in this field. Calvo and D’Mello [21] provided an overview of recent progress in AC, focusing on affect detection. This highlights the need for an integrated examination of emotional theories from multiple disciplines to develop effective practical AC systems. Wang et al. [22] introduced the multimodal emotion database (MED4), encompassing EEG, photoplethysmography, speech, and facial images for emotion recognition research, demonstrated the superiority of EEG signals in emotion recognition, and proposed fusion strategies that combine speech and EEG data to significantly enhance accuracy and robustness.

Tsiourti et al. [23] examined how incongruity in emotional expressions displayed by humanoid robots affects their recognition and response. Their research underscored the negative impact of such incongruence on the robot’s appeal and credibility. A retrospective examination of previous studies in this field is necessary to comprehend the role of cross-sensory modalities in EEG-based emotion recognition. A critical limitation of applying the EEG-based aBCI is that the required system training and test data are highly sensory-dependent. Additionally, the current EEG-based aBCI system can only be based on one type of sensation from emotion simulation. For example, if an EEG-based aBCI is trained using EEG data from auditory stimuli, its operation is limited to auditory stimulation.

Similarly, its operation is limited to visual stimulation if it is trained using EEG data from visual stimuli. If an EEG-based aBCI is trained using EEG data from audio-visual stimuli (e.g., video stimuli), its operation requires audio-visual stimulation. In real-world applications, emotional stimuli are inherently multimodal [24]. Video stimuli contain auditory and visual modalities, resulting in a compound multimodal representation. When exposed to a partial sensory component of a multimodal emotional stimulus, individuals naturally perceive the same emotional category [25]. For instance, when individuals receive the audio, the visual, or both (audio-visual) components for the same video stimulus, the emotional category should be the same and identifiable using the EEG-based aBCI. Nevertheless, owing to sensory differences, the features extracted from EEG data can vary for different sensory modalities, even if they originate from the same stimulus source (e.g., the same video material). The key challenge in this study was effectively extracting features and mitigating the differences between different sensory modalities in EEG data. The cross-sensory EEG emotions were lacking in earlier research. However, the cross-sensory theme was highly related to transfer learning in EEG-based emotion recognition. This is valuable for tracing the previous comprehensive research on transfer learning in EEG-based emotion recognition. Existing transfer learning research has mainly focused on cross-subject, cross-session, and cross-dataset. Cimtay et al. [26] introduced a novel multimodal emotion recognition system using facial expressions, galvanic skin response (GSR), and electroencephalogram (EEG) data, achieving high accuracy rates and surpassing reference studies in subject-independent recognition. Li et al. [27] presented the self-organized graph neural network (SOGNN) for cross-subject EEG emotion recognition, achieving state-of-the-art performance by dynamically constructing graph structures for each signal. CLISA (Contrastive Learning method for Inter-Subject Alignment), a method developed by Shen et al. [28], leverages contrastive learning to minimize inter-subject differences and improve cross-subject EEG-based emotion recognition by extracting aligned spatiotemporal representations from EEG time series. Domain adaptation with adversarial adaptive processes has recently gained increasing attention, achieving state-of-the-art performance. Wang et al. [29] proposed a multimodal domain adaptive variational autoencoder method by learning shared cross-domain latent representations and reducing distribution differences, demonstrating superior performance in emotion recognition with small labeled multimodal data. Guo et al. [30] proposed a multi-source domain adaptation with a spatiotemporal feature extractor for EEG emotion recognition, effectively reducing cross-subject and cross-session, demonstrating its powerful generalization capacity. He et al. [31] proposed a method that combines temporal convolutional networks and adversarial discriminative domain adaptation for EEG-based cross-subject emotion recognition, effectively addressing the domain-shift challenge. Sartipi and Cetin [32] proposed an approach that combined transformers and adversarial discriminative domain adaptation for cross-subject EEG-based emotion recognition, achieving improved classification results for valence and arousal.

**Figure 1:**
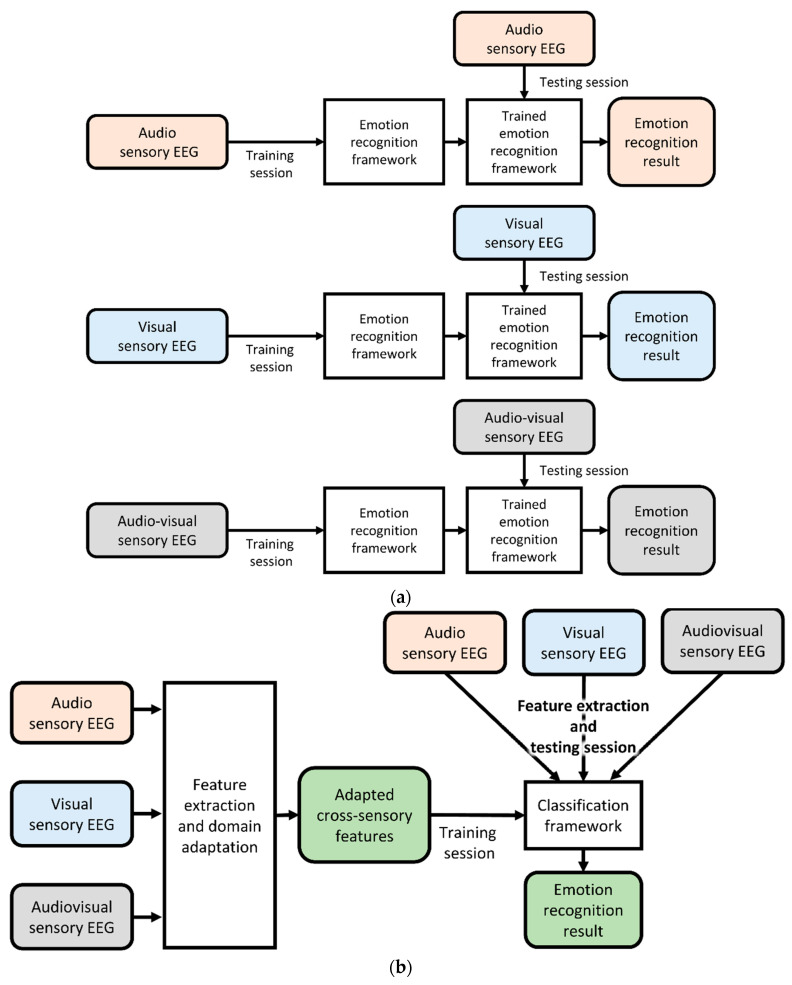
Demonstration of previous EEG-based aBCI (**a**) and the proposed cross-sensory EEG-based aBCI (**b**). The stimuli source was video stimulation.

This study aimed to effectively extract features and mitigate the differences in EEG data between sensory modalities using domain adaptation techniques. The previous EEG-based aBCI and proposed cross-sensory EEG-based aBCI are illustrated in Figure 1. Emotional EEG data can be used to train the aBCI system regardless of the sensory modality. Establishing a robust emotion recognition framework is essential to address this issue.

Hence, inspired by previous research on general emotion recognition, transfer learning-based emotion recognition, and domain adversarial adaptation, we conducted experiments to collect cross-sensory emotion EEG data with a video as the source stimulus. Audio-, visual-, and audio-visual sensory-inspired EEG data were obtained from 20 participants. Subsequently, filter bank adversarial domain adaptation Riemann methods (FBADR) have been proposed for cross-sensory EEG emotion recognition. Specifically, the proposed methods use filter banks and Riemann methods for feature extraction. An adversarial domain adaptation method inspired by conditional Wasserstein generative adversarial networks was explored for domain adaptation. The classification was conducted using an ensemble of SVMs with a meta-classifier. Experimental results from the proposed FBADR revealed its state-of-the-art performance in cross-sensory emotion recognition with high robustness. This study could also be recognized as pioneering research in cross-sensory emotion recognition with a video stimulus as multimodal emotion stimulation using two categories of emotion: pleasure and displeasure. The results of this study can be further applied to EEG-based aBCI for theoretical analysis and practical applications. The table of abbreviations, their full names, and usages are provided in Supplementary Material Table S1.

## 2. Materials and Methods

### 2.1. Data Acquisition Paradigm

Cross-sensory EEG emotion recognition experiments were conducted using EEG data from self-designed multimodal emotion-stimulation experiments [33]. Twenty emotion videos—ten of pleasure and ten of unpleasure—sourced from the New Standardized Emotional Film Database for Asian Culture [34] were explored as multimodal emotion stimulation for inducing cross-sensory EEG emotion data during the experiment. Self-assessments were performed after stimulation in each trial. The participants were twenty healthy participants with no previous mental or physical injury and currently native Chinese speakers. In the experiment, two computers were utilized; one was dedicated to controlling the stimulation process, while the other was used for recording EEG data and manually inspecting the recorded information. As the stimulation control window could potentially interfere with the window for EEG data recording, we employed two separate computers for these tasks to ensure the smooth progress of the experiment.

**Figure 2:**
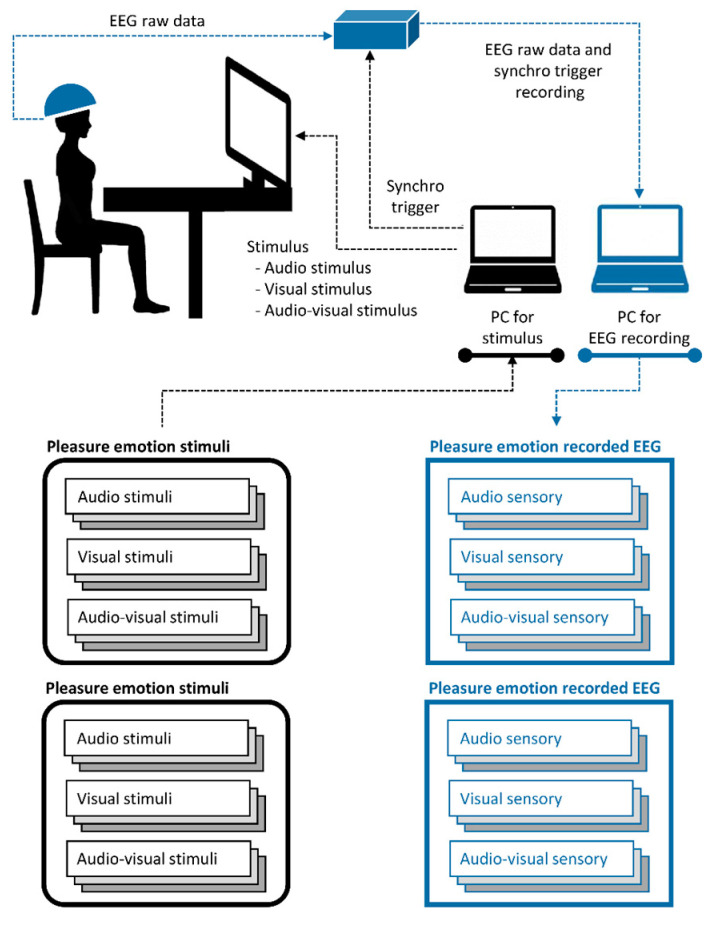
Description for cross-sensory EEG emotion data acquisition.

There are three stimulus modalities for inducing their respective sensory modalities: audio, visual, and audio-visual. The audio stimulus modality provided audio information from the emotion videos, the visual stimulus modality provided visual information from the emotion videos, and the audio-visual modality provided audio and visual information from the emotion videos. Hence, we had two types of emotions (pleasure or displeasure) and three stimulus modalities (audio, visual, and audio-visual) for inducing EEG data. The collected cross-sensory emotion EEG data can be described as follows: pleasure EEG (audio/visual/audio-visual sensory) and unpleasure EEG (audio/visual/audio-visual sensory). The experimental setup is illustrated in Figure 2. Twenty healthy participants were recruited for the experiment. The experimental details and data validation have been described in previous studies [33]. Detailed information on the cross-sensory EEG emotion data is presented in Table 1.

### 2.2. Data Preprocessing

**Figure 3:**
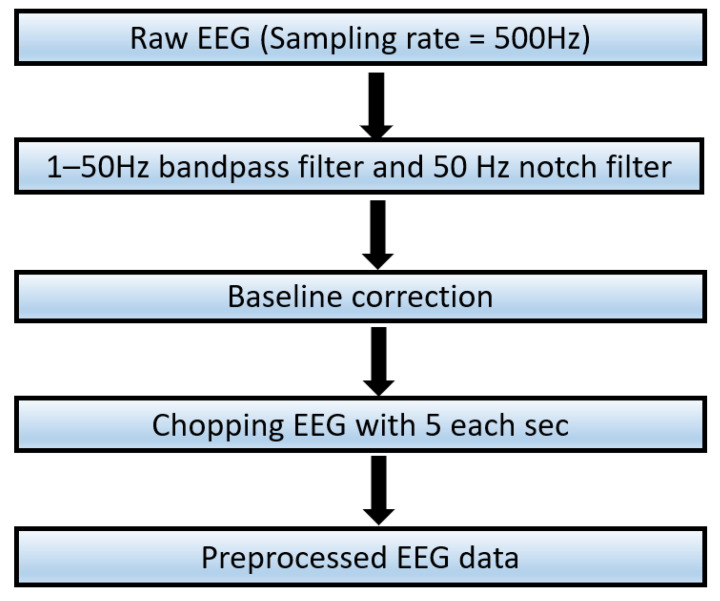
Workflow for EEG data preprocessing.

The data preprocessing is described in Figure 3.

Preprocessing cross-sensory EEG emotion data for cross-sensory emotion recognition involved the following steps. An applied finite impulse response (FIR) bandpass filter facilitated EEG signal extraction between 1 Hz and 50 Hz [35,36], with a subsequent notch filter employed to eliminate 50 Hz mains power interference. Baseline correction was conducted within the initial 1000 ms before stimulation onset. The primary focus of the investigation was the EEG data spanning 0–30 s during stimulation, utilizing a 5 s window [37,38,39]. A total of 7200 samples were analyzed, comprising 60 samples per participant for each of the six cross-sensory EEG data types, each sample containing 2500 temporal features derived from 500 × 5 data points. The EEG data consisted of 32 channels, referencing FCz. Excluding FCz, 31 channels were utilized for further framework implementation. The above preprocessing steps were executed by the MNE 1.2.1 [40] library in Python 3.6.

## 3. Methodology

### 3.1. Filter Bank Riemannian Feature Extraction

Emotional EEG signals contain information of various frequency ranges. By categorizing the signals into different frequency bands, the features within each band can be better captured. The frequency bands are associated with different neural activities related to emotional states. Therefore, separating them helps extract emotion-related features accurately. Different frequency ranges of EEG signals are associated with specific neural activity. For instance, the low-frequency range (1–4 Hz) is often associated with relaxation and resting, whereas the high-frequency range (30–50 Hz) is associated with attention and arousal states [41]. Dividing the signals into different frequency bands allowed for a more accurate representation of the influence of different neural activities on emotional states. The most frequently used frequency bands were 1–4 Hz (delta), 4–8 Hz (theta), 8–13 Hz (alpha), 13–30 Hz (beta), and 30–50 Hz (Gamma) [42]. In this study, we further divided the beta into two sub-bands:13–20 Hz (Beta 1) and 20–30 Hz (Beta 2), to better distinguish different neural activities and EEG signal characteristics [43,44]. In the beta band, the lower frequency range (13–20 Hz) is typically associated with attention, cognitive processing, and emotion regulation, whereas the higher frequency range (20–30 Hz) tends towards motor control and perception [45]. Different neural activities and features can be captured more precisely by separating them. Therefore, we used IIR bandpass filters to divide the preprocessed 1–50 Hz EEG signals into six sub-bands: 1–4 Hz (Delta: band 1), 4–8 Hz (Theta: band 2), 8–13 Hz (Alpha: band 3), 13–20 Hz (Beta 1: band 4), 20–30 (Beta 2: band 5), and 30–50 Hz (Gamma: band 6).

The Riemannian space method has good generalizability in transfer learning. By learning Riemannian features from the source domain, transfer learning can be performed in the target domain, reducing the sample requirements and improving the classification performance [46]. Riemannian space methods can capture shared structures and features between source and target domains, enabling knowledge and model transfer.

Extracting Riemannian features from EEG signals involves computing the covariance matrix (CM) in SPD form [47]. EEG signals are typically represented as multi-dimensional time-series data, in which each observation at a given time corresponds to a voltage recorded at different electrode locations. Transforming these time series into a matrix representation is necessary for feature extraction and classification. We can capture the correlation and coactivity between different electrodes in the EEG signals by computing the covariance matrix. The covariance matrix is a symmetric positive-definite, describing the spatial covariance relationships between the different electrodes. Hence, the dimensions of the covariance matrix are related to the number of electrodes. Therefore, computing the covariance matrix in its SPD form is necessary for the Riemannian feature extraction of EEG signals. This allows the transformation of EEG signals into points on the Riemannian manifold and facilitates feature extraction and classification analysis using the geometric structure of the manifold.

x and y represent EEG signals from the two channels, with N as the window length or time point of the signals. Preprocessing EEG signals had a signal mean of zero by filtering the direct components; therefore, the CM between both channels can be obtained using Equation (1).

(1) $$Cov\left(x,y\right)=\frac{1}{N}\sum_{i=1}^{N}\left(x_{i}y_{i}\right)$$

Let the preprocessed EEG trials be $X_{i}\in R^{K*N}$, i=1,…,t; K is the number of electrodes, t is the total number of trials. The corresponding CM for $X_{i}$ can be determined using Equation (2):

(2) $$CM_{i}=\frac{1}{N-1}X_{i}X_{i}^{T}$$

T is the transpose operation of the matrix.

We used the oracle approximating shrinkage [48] regularization method to ensure that all covariance matrices were regularized (in the symmetric positive-definite form). The shape of the CM corresponding to each EEG trial depended on the number of signal channels. There were K signal channels for each EEG trial; thus, the shape of the CM was (K, K). The number of rows and columns in this matrix equals the number of signal channels, representing the correlations and variances between each channel. The series of SPD CMs are denoted as $sym^{+}=\{CM\in R^{K\times K},x^{T}CMx>0,CM=CM^{T},\forall x\in R^{K}$ and x is a non-zero vector.

CMs naturally belong to the Riemannian manifold rather than the Euclidean space [48]. Transformative operations are required to apply operations suitable for Euclidean space.

**Figure 4:**
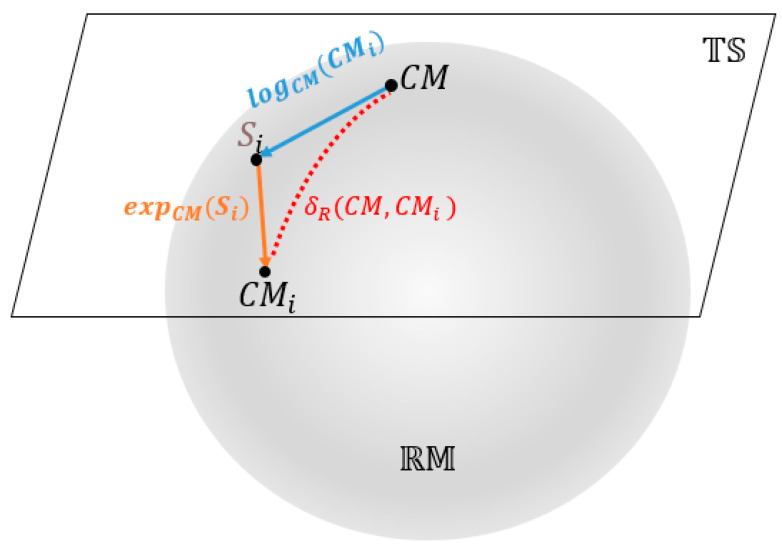
Riemannian manifold and tangent space.

However, using the original CMs as features for classification is challenging because they reside on the Riemannian manifold RM, not in Euclidean space. The features extracted from the Riemannian tangent space have better discriminative properties and are easier to handle than the original Riemannian space features [49]. This is primarily due to their linear properties and enhanced discriminability. In tangent space, traditional linear methods such as the Euclidean distance and linear classifiers can be directly applied, as the space is linear, and linear operations have simpler expressions. Additionally, the projection operations in the tangent space highlight the differences between the sample classes, improving feature discriminability. In contrast, the original Riemannian space may have smaller differences between sample classes, leading to lower discriminability of the features. We will utilize the transformation from the $CM\in sym^{+}$ at the Riemannian space—RM of CMs to the tangent space—TS to address this issue. Figure 4 illustrates the two-dimensional Riemannian manifold and tangent spaces.

Based on singular value decomposition, the operation logm is denoted as logarithm of a matrix [50], and the Riemannian space point $CM_{i}$ to the tangent space can be described as in Equation (3):

(3) $$S_{i}=log_{CM}\left(CM_{i}\right)=CM^{\frac{1}{2}}\left(logm\left(CM^{-\frac{1}{2}}CM_{i}CM^{-\frac{1}{2}}\right)\right)CM^{\frac{1}{2}}$$

Inversely, the projected tangent space points can be transferred into the Riemannian space using the exponentialmap, denoted as expm, which can be described as in Equation (4):

(4) $$CM_{i}=log_{CM}\left(S_{i}\right)=CM^{\frac{1}{2}}\left(expm\left(CM^{-\frac{1}{2}}S_{i}CM^{-\frac{1}{2}}\right)\right)CM^{\frac{1}{2}}$$

The distance between CM and $CM_{i}$ are denoted as $\delta_{R}\left(CM,CM_{i}\right)$ in Figure 4.

Features extracted from the Riemannian tangent space have better discriminative properties and are easier to handle than the original Riemannian space features. This is primarily due to their linear properties and enhanced discriminability. The Euclidean distance and linear classifiers can be directly applied in the tangent space because the space is linear, and linear operations have simpler expressions. Additionally, the projection operations in the tangent space highlight the differences between the sample classes, improving feature discriminability. In contrast, the original Riemannian space may have smaller differences between sample classes, with lower feature discriminability. The features of this study are the tangent-space CMs. Therefore, from Equations (3) and (4), the original Riemannian space $CM_{i}^{RM}=CM_{i}$ can be transformed into tangent-space $CM^{TS}$—using Equation (5), known as the log-Euclidean mean covariance [17].

(5) $$CM^{TS}=expm\left[\frac{1}{t}\sum_{i}^{t}logm\left(CM_{i}^{RM}\right)\right]$$

**Figure 5:**
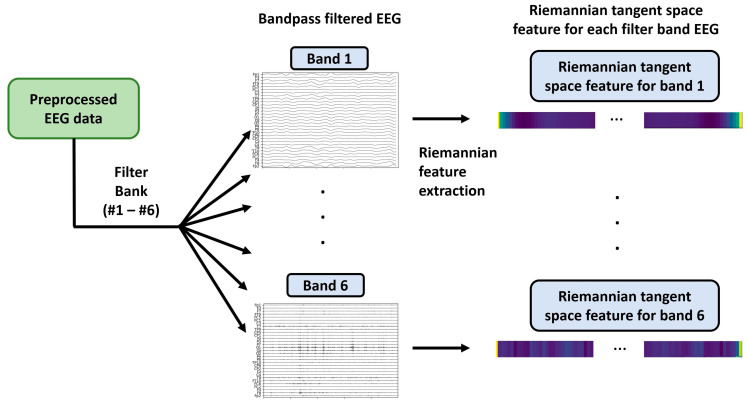
Workflow for filter bank Riemannian feature extraction.

The set of Riemannian tangent features in each frequency band could be obtained using $CM_{i}^{TS}\in R^{K\times K},where\;i=1,\ldots ,\;t$. There are further domain adaptation and classification requirements; therefore, we flattened each trail feature from the two-dimensional Riemannian tangent features—$CM_{i}^{TS}\;\in R^{K\times K}$ as $CM_{i}^{TS\;flatten}\in R^{F\times 1},F=K*K$. The workflow for feature extraction using the filter bank and Riemannian methods is illustrated in Figure 5.

### 3.2. Adversarial Domain Adaptation

This study mainly focused on Riemannian tangent space features for feature interpolation and analysis, which can be obtained as described in Section 3.2. Features from one of the three sensory modalities can be recognized as the target domain to fulfill the cross-sensory emotion recognition requirements. Thus, the features of the remaining two sensory modalities were recognized as the source domains, for instance, when the features from audio sensory modalities constituted the target domain. Conversely, those from the visual and audio-visual sensory modalities constituted the source domain. Reducing the discrepancy between the source and target domains and making the source-domain features proximal to those of the target domain are crucial to domain adaptation. Therefore, we employed an adversarial domain adaptation approach to achieve domain adaptation from the source to the target domain.

Adversarial domain adaptation evolved from generative adversarial networks (GANs) [51] and addressed domain adaptation challenges. GANs consist of generator and discriminator networks trained in an adversarial manner to generate realistic data samples. Adversarial domain adaptation leverages the adversarial training concept of GANs to address domain adaptation, which involves transferring knowledge from a source domain with labeled data to a target domain. Adversarial domain adaptation has two key components: a feature extractor and a domain discriminator. The feature extractor learns the shared representation of the input data, whereas the domain discriminator differentiates the source from the target domains based on the learned features. Adversarial domain adaptation involves minimizing the distribution discrepancy between the source and target domains through a feature extractor, making the features more similar across domains. Simultaneously, the domain discriminator maximizes the distribution discrepancies to distinguish the domains. Adversarial training helps feature extractors generate domain-invariant representations, enabling knowledge transfer and performance improvement in the target domain. Hence, adversarial domain adaptation builds upon the adversarial training concept of GANs and extends it to address domain adaptation challenges. Minimizing feature distribution discrepancy and maximizing domain discrimination facilitate knowledge transfer from a labeled source domain to an unlabeled target domain, enabling adaptation and improved performance in the target domain.

The original GAN algorithm was first introduced in 2014 [51]. The main idea was a minimax process with two elements: generator—G and discriminator—D. The generator can create real-like fake data by inputting random noise into the generator. The discriminator distinguishes between the generated real-like fake data and the real data. This minimax process aims to train the generator to produce real-like fake data to trick the discriminator into recognizing the generated data as real. In the domain adaptation field, the feature adaptor replaces the original generator by inputting the source-domain features into the adaptor to generate source-domain features that are target domain-like from the domain adaptor. The proposed adversarial domain adaptation framework comprises a feature adaptor and a discriminator.

With the loss function L, the entire minimax process based on the original GAN for adversarial domain adaptation can be described as follows:

(6) $$\;L_{\min_{AD}{\;\max}_{DS}\;}=E_{x_{a}\sim X_{a}}\left[\log(DS\left(x_{a}\right))\right]+E_{r\sim z_{r}\left(r\right)}\left[\log(1-DS\left(AD\left(r\right)\right))\right]$$

In Equation (6), $X_{a}$ represents the target-domain features and $DS\left(x_{a}\right)$ represents the discriminator for calculating the x probability of the target domain distribution. The input source-domain features are $z_{r}$; AD(r) is an adaptor used with the input r source-domain features, outputting the targeted domain-like features.

**Figure 6:**
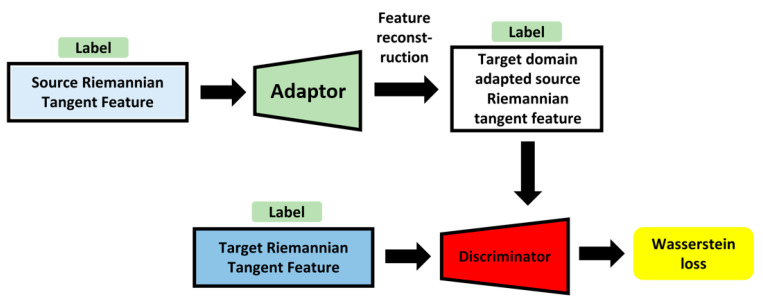
Illustration for proposed adversarial domain adaptation framework.

The features from the target and source domains were extracted using the Riemannian tangent space methods. Previous research has indicated that the original GAN loss is vulnerable to collapse during training sessions. Meanwhile, the emotion-corresponding labels in this study were pleasure and displeasure, regardless of sensory modality. Feature adaptation can fit each emotion label corresponding to the target domain rather than the emerged target domain. Inspired by Wasserstein generative adversarial networks [52] and adding label information as conditions, the proposed adversarial domain adaptation framework is indicated in Figure 6.

The detailed structures and parameters of the proposed domain adaptor and discriminator are presented in Table 2 and Table 3, respectively.

The min-max problem for the proposed adversarial domain adaptation process is described by Equation (7).

(7) $$\;Lw_{\min_{AD}{\;\max}_{DS}\;}=-E_{x_{a}\sim X_{a}}\left[DS\left(x_{a}|y_{a}\right)\right]+E_{r\sim z_{r}\left(r\right)}\left[DS(r|y_{r})\right]+\lambda E_{\hat{x}\sim \hat{X}}[{\left(\nabla_{\hat{x}}DS\left(\hat{x}\right)y_{r}{)}_{2}-1\right)}^{2}]$$

The last term in Equation (7) is a penalty element. The data point was sampled from a straight line between the target domain $X_{a}$ and the adapted source domain $X_{AD}$ is $\hat{X}$, where $x_{a}$ denotes the data from $X_{a}$. The hyperparameter λ controls the trade-off between the target domain and the gradient penalty.

The detailed parameters and functional components of the adaptor are listed in Table 2, with one dense layer, one reshaped layer, and two convolutional layers. The leaky ReLU function had a good performance in GANs [53,54]. Therefore, we chose a leaky ReLU activation layer, followed by a dense layer and the first convolutional layers. We chose a tanh activation layer attached to the last deconvolutional layer for the final adaptor output. In each convolutional layer, batch normalization was introduced to increase the solution speed of the gradient descent and avoid overfitting.

The detailed parameters and functional parts, specifically explaining each layer, are described in Table 3. A leaky ReLU activation layer followed each convolutional layer, and batch normalization was performed after each convolutional layer. The final output of the discriminator uses the sigmoid function. The kernel size for each convolutional layer in the adaptor was three, and the convolutional layer in each adaptor was two, based on previous GAN-related studies [55,56,57]. After implementing the proposed adversarial domain adaptation networks, the adapted source-domain features could be obtained, and the target and source-domain features were cross-sensory features used together to train the classifiers.

### 3.3. Classification Strategy

**Figure 7:**
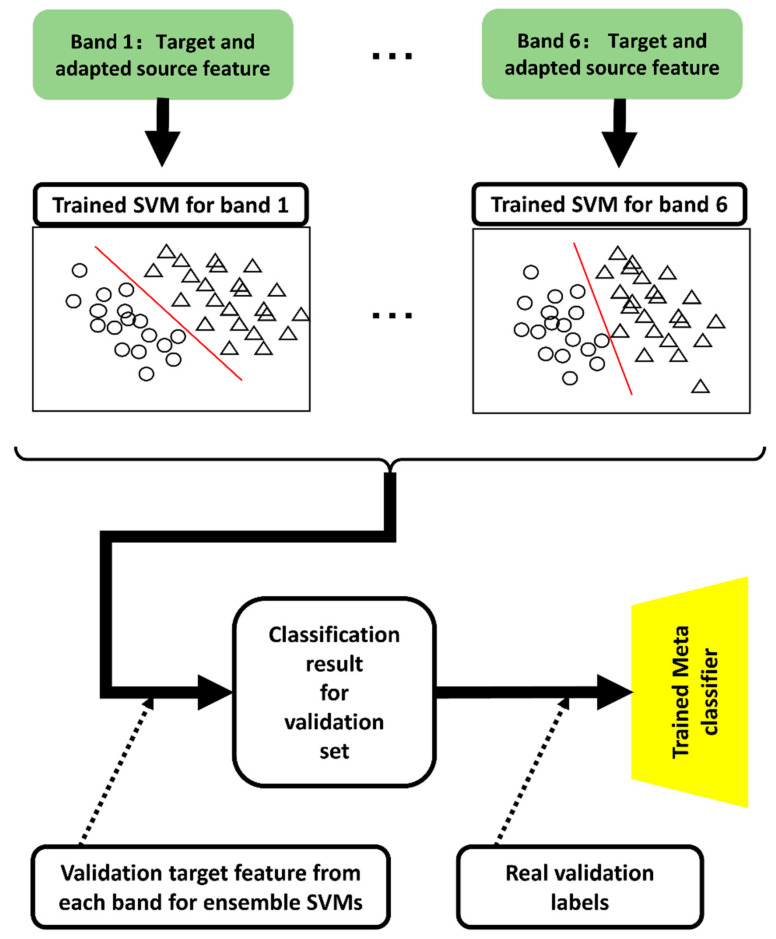
Description for ensemble SVM classification strategy.

After executing domain adaptation, the adapted source and target-domain features were used with the corresponding bands. The adapted source and target-domain features are recognized as Riemannian domain target features; therefore, the SVM with polynomial kernels had a considerable discriminative performance for these specific features [58]. Therefore, we explored polynomial kernel SVMs for each band feature to obtain trained SVM classifiers for emotion recognition. Moreover, six trained SVMs corresponded with the six bands to ensemble the six SVMs’ results into final emotion recognition outputs. The proposed classification strategies are illustrated in Figure 7.

The target and adapted source features for each band constituted the training data corresponding to each SVM classifier. After the training session, six SVMs were trained for each of the six bands.

Each predicted output with the trained SVMs is identical to each label. Therefore, the transformational relationship between the six SVM outputs and the final classification result cannot be adjusted using the training set directly. Inspired by the blending of stacked methods for ensemble learning [59], we introduced a validation set for blending the transformative relationship between the six SVM outputs into the final classification result.

A set of validation data was explored to ensemble the six SVMs. A set of predicted validation data labels was obtained by inputting the validation target features from each band into each corresponding SVM. The predicted validation data labels are denoted as $L_{i}^{bandj},j=1,\ldots ,\;6,\;i=1\ldots n_{v}$; $n_{v}$ is the number of trials for the validation set. The $L_{i}^{bandj}$ is the input feature for the meta-learner and the real validation labels serve as the input label information for the meta-classifier.

Two classifiers were used for the blending methods. The basic classifiers obtained the predicted multiple outputs from the original input features, and a meta-classifier for receiving multiple outputs from basic classifiers and providing the final classification results. In this study, the basic classifiers were SVMs. A logistic regression (LR) model was selected as the meta-classifier for the final susceptibility prediction because of the effectiveness of simple linear models [60]. Therefore, LR was trained to ensemble the outputs from the six SVMs. In the test session, emotion recognition results for the test data were obtained by inputting the filter bank Riemannian feature from the target and adapted source features for the proposed trained SVM-meta learner framework. Based on Section 3.1, Section 3.2 and Section 3.3, the training process of proposed FBADR could be described as Algorithm 1.
**Algorithm 1** Training process of proposed FBADR1. Filter bank     Determine sub-bands EEG signals banks $X_{i|bandj}\in R^{K*N}$, j =1…6 from filter bank method. 2. Riemannian method     Compute the covariance matrix for each sample $CM_{i|sub}$ from Equation (2).     Compute the $CM^{TS}$ as Riemannian tangent space features from Equation (5). 3. Adversarial domain adaptation     Determine adapted source-domain feature $X_{AD}$ from Equation (7). 4. Ensembled SVM classifier training     Train SVMs with target and adapted source feature and labels from band 1 to band 6.     Determine $L_{i}^{bandj}$ by inputting the validation target features into trained SVM correspondingly.     Train meta learner with $L_{i}^{bandj}$ and real validation labels.

## 4. Experimental Results and Discussion

### 4.1. Experimental Description

**Figure 8:**
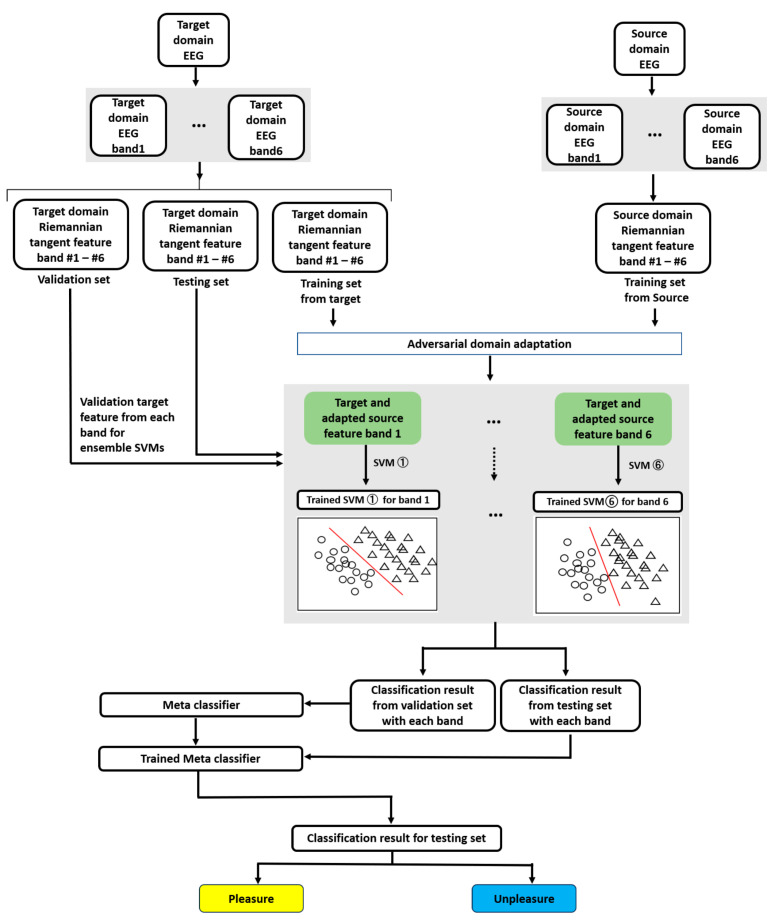
Workflow for entire cross-sensory EEG emotion recognition based on proposed FBADR.

The experiment can be described in three sessions: feature extraction, adversarial domain adaptation, and classifier training and validation, as illustrated in Figure 8. For example, the target domain EEG was considered audio sensory, and the source-domain EEG as visual and audio-visual sensory. All experiments were executed on Windows 10 and Python 3.6, with an Intel i7-10875H CPU and an NVIDIA RTX, the 2080s GPU.

Feature extraction was performed using the MNE 1.2.1, SciPy 1.5.4, and NumPy 1.23.5 libraries. In the feature extraction session, the target domain EEG first underwent six bandpass filters to obtain a target domain EEG with six bands. Then, based on the well-known transfer learning metric for cross-subject on EEG decoding—Leave-One-Subject-Out [61]—we chose “Leave-One-Sensory-Out.” Specifically, we used one sensory modality as the target domain and the other two as source domains for each subject. The target domain data were split into a training, validation, and test set of 60%, 20%, and 20%, respectively, coordinated with each band’s EEG data. The training, validation, and testing sets were used for Riemannian feature extraction. The source-domain EEG served only as training data and was passed through filter banks and Riemannian feature extraction.

Adversarial domain adaptation frameworks were built and executed based on TensorFlow1.13.1; the Adam optimizer [62] with Learning rate=0.001 was used for network optimization. In the adversarial domain adaptation session, the source-domain features were adapted using the proposed adversarial domain adaptation methods coordinated with the extracted features from the target domain training set. The target and adapted source features from each band were obtained from an adversarial domain adaptation session. For each target sensory modality subject, 100 training epochs with a batch size of 32 were used to train the framework. After completing the training session, a trained adaptor was used to generate the adapted source domain features.

In the classification session, the frameworks were realized using Scikit-learn to build the SVM and meta-classifier LR. Using GridSearchCV in subjects 01, 02, and 03, SVM parameters were adjusted as C=0.001, kernel=‘poly’, gamma=10, and the LR-based meta classifier was used under the default parameters. In training and validating the classification session, the target and adapted source features from each band served as training features for each SVM. Emotion labels (pleasure or displeasure) constituted the training label information. Subsequently, six SVMs were trained through stacked ensemble methods using LR classifiers trained by inputting the classification results of the six SVMs from the validation set of target-domain features as input features. The real label information of the validation set of target-domain features was used as training label information. An ensemble of six final classification outputs was built. Finally, the testing set features were used with six trained SVMs to obtain the classification outputs, which underwent the trained LR, and the classification results from the testing set were obtained. We used five-fold cross-validation methods to obtain the final classification results for each subject in each target sensory modality. Specifically, for five-fold cross-validation, each subject’s target-domain features were randomly shuffled while maintaining the proportion of the label information. The shuffled data were divided into five equal subsets. For each subset, three of the remaining four constituted the training set, and one was the validation set. This subset was used as the test set. The allocated training, validation, and test sets were used for domain adaptation and classification, and classification results were obtained. This process was repeated five times until each subset was used as the test set. Therefore, all the subjects’ target-domain features were included in the model evaluation.

### 4.2. Adversarial Riemannian Methods Validation

**Figure 9:**
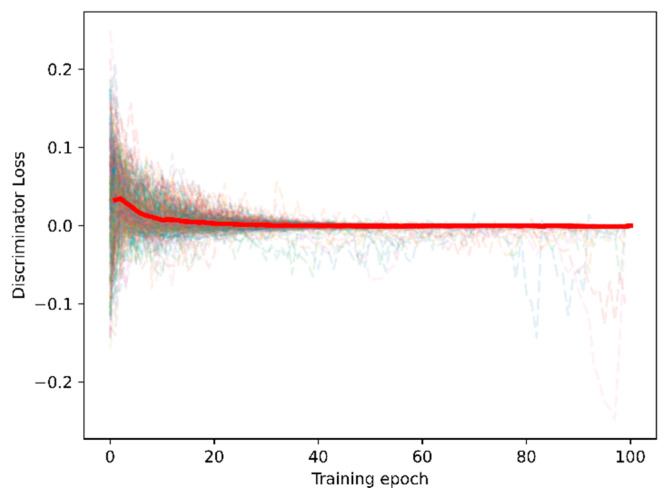
Loss curve under training sessions of the adaptor and discriminator.

The proposed adversarial domain adaptation method aimed to align the source-domain features to the target domain. The training process loss of the adaptor and discriminator for 20 subjects × 3 sensory modalities × 6 filter banks = 360 training sessions; the loss during the training session is indicated in Figure 9 to illustrate the validation of the proposed adversarial domain adaptation.

We fitted and highlighted the average loss curve for all 360 training sessions. Figure 9 indicates that the adaptor and training losses converged to zero under 100 training epochs. Hence, the purpose of minimum-maximum training for domain adaptation was confirmed.

**Figure 10:**
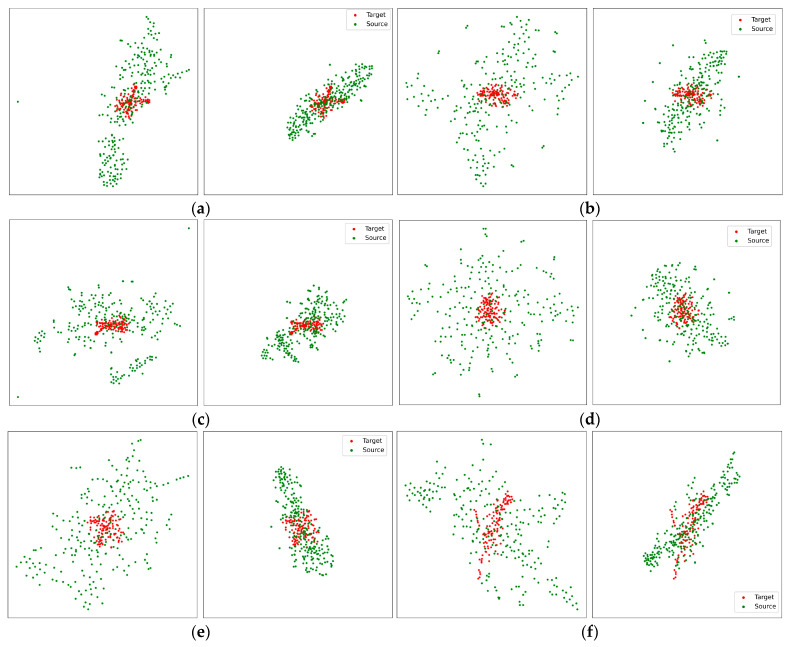
Visualization of target-domain features and original/adapted source-domain features. Red points were target domain features; green points were source domain features. For (**a**–**f**), The left subfigures are target domain features and original source domain features, the right subfigures are target domain features and adapted source domain features. From the target domain features (**a**): sub01; audio; 1–4 Hz, (**b**): sub04; audio-visual; 4–8 Hz, (**c**): sub17; visual; 8–13 Hz, (**d**): sub11; visual; 13–20 Hz, (**e**): sub05; audio; 20–30 Hz, (**f**): sub10; audio-visual; 30–50 Hz, the source domain features were the corresponded subject, filter banks and the rest of two sensory conditions.

Moreover, we compared the target and original/adapted source-domain features to investigate the domain alignment from the proposed adversarial domain adaptation methods by visualizing two-dimensional t-stochastic Neighbor Embedding [63], as illustrated in Figure 10.

Red points indicate the target-domain features; green points represent the source-domain features. For (a), (b), (c), (d), (e), and (f), the left subfigures are the target and the original source-domain features, and the right subfigures are the target and adapted source-domain features. Figure 10 reveals the success of domain adaptation by comparing the target and original/adapted source-domain features. Via the proposed methods, the source-domain Riemannian features have been adapted with the target domain Riemannian features.

### 4.3. FBADR Emotion Recognition Results

**Figure 11:**
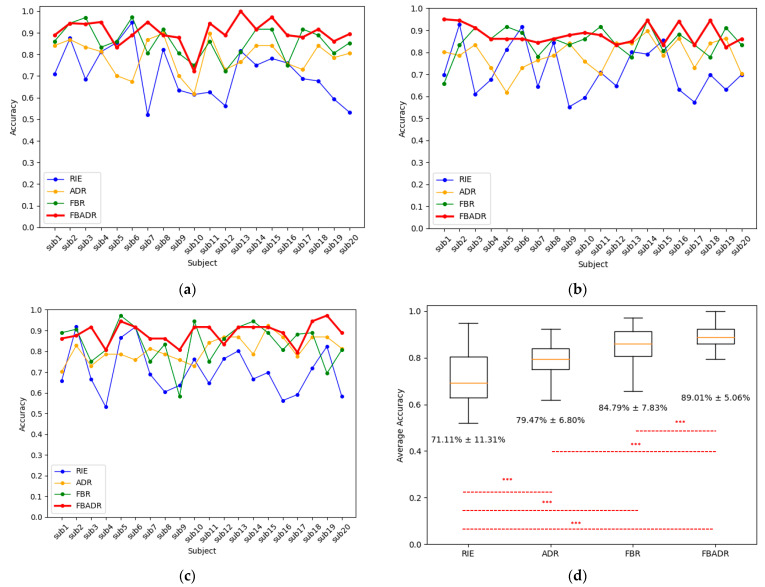
Cross-sensory emotion recognition results from RIE, ADR, FBR, and FBADR. The classification results for each subject with the target domain as audio, visual, and audio-visual are (**a**–**c**), respectively; (**d**) is the average accuracy of 20 subjects and three sensory modalities, the text in (**d**) indicate the average±std of the accuracies.

The cross-sensory emotion recognition results were obtained using the proposed FBADR methods (Figure 11). Meanwhile, for a comparative study, the classification results of Riemannian methods (RIE), which use Riemannian feature extraction without filter banks and domain adaptation, adversarial domain Riemannian methods (ADR), filter bank Riemannian methods (FBR), and Riemannian feature extraction with the proposed adversarial domain adaptation, were used. RIE and ADR did not have multibank features; therefore, we used only SVM for classification, and the FBR classification strategy was the same as that of the proposed FBADR. The emotion labels with the proportion of pleasure: unpleasure=1:1 for binary emotion recognition; thus, the accuracy could be utilized for model evaluations. The accuracy can be determined using Equation (8):

(8) $$CC=\frac{TP+TN}{TP+FN+TN+FP}$$

We classified pleasure as true positive (*TP*) and classified displeasure as true negative (*TN*), false positive (*FP*), or false negative (*FN*).

The proposed FBADR methods had the best classification results in cross-sensory emotion recognition, with an average accuracy of 89.01%±5.06% (Figure 11). RIE, ADR, and FBR had average accuracies of 71.11%±11.31%, 79.47%±6.80% and 84.79%±7.83%, respectively. Additionally, the average computational cost in training and test sessions for RIE, ADR, FBR, and FBADR was 0.03 s, 1.22 s, 0.21 s, and 7.87 s for each subject. Compared with RIE, the FBADR mainly increased computational cost due to the neural networks-based domain adaptation training with sub-bands of approximately six-times repetitive operations, resulting in a better EEG decoding result. Figure 11a–c reveal that FBADR performed best with audio, visual, and audio-visual sensory modalities as the target domains. One-way analysis of variance (ANOVA) was used to determine the significant differences between each method, and a *p*-value of < 0.001 for RIE-FBR, RIE-ADR, RIE-FBADR, ADR-FBADR, and FBR-FBADR pairs are denoted as *** in Figure 11d. The filter bank and adversarial domain adaptation methods can also improve cross-sensory emotion recognition.

Meanwhile, in order to assess the impact of various SVM kernels on the performance of the proposed FBADR method, we incorporated different types of kernels including polynomial, linear, and radial basis function (RBF) kernels into the SVM framework. Specifically, for the implementation of FBADR, we utilized SVMs with polynomial, linear, and RBF kernels, setting the parameters as C = 0.001 and gamma = 10. The outcomes obtained from employing these three distinct kernels are outlined in Table 4.

The results from Table 4 indicated that the polynomial kernel-based SVM classifier performed best in the Riemannian feature-based classification task [58].

### 4.4. Baseline Methods Comparison

The cross-sensory EEG emotion recognition is an emerging challenge in the EEG aBCI field; therefore, no related studies exist. To obtain a comprehensive performance evaluation, we chose six recently published EEG decoding frameworks for comparative study:

KNN [64], an EEG emotion recognition method that utilized entropy and energy, was calculated as features after being divided into four frequency bands using discrete wavelet transform and a K-nearest neighbor (KNN) classifier. Frequency band features from the Gamma band were used as a baseline.

EEGNET [65], an end-to-end EEG decoding framework based on neural networks, has been widely adopted in emotion recognition, motor imagery, and other BCI fields. ICRM-LSTM [17], a model for EEG-based emotion recognition by combining the independent component analysis (ICA), the Riemannian manifold (RM), and the long short-term memory network (LSTM).

PCC-CNN [13], an EEG Emotion recognition framework with Pearson’s correlation coefficient (PCC)-featured images of channel correlation of EEG sub-bands and the CNN model to recognize emotion.

DANN [66], a transfer learning-based EEG emotion recognition framework with adversarial domain adaptation neural networks for an cross-subject EEG emotion recognition framework.

WG-DANN [67], a transfer learning-based EEG emotion recognition framework, consists of GANs-like components and a two-step training procedure with pre-training and adversarial training with Wasserstein GAN gradient penalty loss for cross-subject EEG emotion recognition framework.

**Figure 12:**
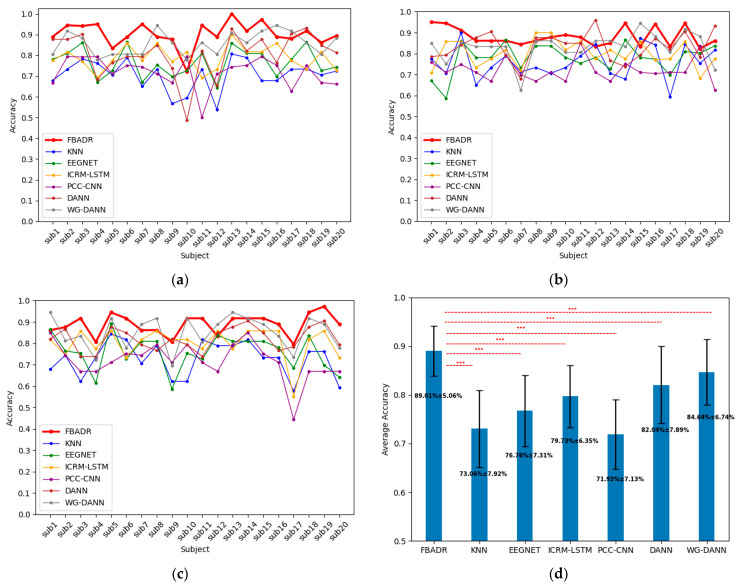
Cross-sensory emotion recognition results from proposed FBADR, KNN, EEGNET, ICRM-LSTM, PCC-CNN, DANN, and WG-DANN. (**a**–**c**) were the classification results for each subject with the target domain as audio, visual, and audiovisual, respectively. (**d**) was the averaged accuracy of 20 subjects and 3 sensory modalities; the text in (**d**) are average±std of accuracy.

All the baseline results were the target domain as one sensory modality and the source domain as the other two for each subject, with the same five-fold cross-validation training strategy as in the proposed FBADR. The input data for the baseline methods were proposed using the workflow described in Section 2.2. The original algorithms from the baseline methods did not contain a domain adaptation process (KNN, EEGNET, ICRM-LSTM, PCC-CNN), and the training data were used directly as original features from the source and target domains. The baseline methods only required the training and testing sets; therefore, the training data we used comprised 80% of the target and all of the source-domain data. The testing data included 20% of the target domain data, implemented with five-fold cross-validation, similar to the proposed FBADR classification strategy. As EEG emotion recognition mainly focuses on improving the classification accuracy of EEG-based emotion recognition BCI systems, we compared the classification accuracy of the cross-sensory EEG data for the proposed FBADR and baseline methods. The classification accuracy of the baseline comparison is presented in Figure 12. The EEGNET was reimplemented from https://github.com/vlawhern/arl-eegmodels (accessed on 15 June 2023), and the rest of the baseline methods were reimplemented based on original papers.

Figure 12 reveals that the proposed FBADR method had the best classification results in the context of cross-sensory emotion recognition compared to the baseline methods, with an improvement of approximately 5% in average accuracy of the best performance of the baseline methods and a lower standard deviation. A one-way ANOVA was used to determine the statistical difference between each method, and a p−value of<0.001 for the pairs of FBADR to the six baseline methods is denoted as *** in Figure 12d. The average accuracy and statistical difference between the proposed FBADR and baseline methods in the experiments indicated the viability of the proposed FBADR method for cross-sensory emotion recognition.

By employing the filter bank ensemble approach, it is possible to decompose the original signal into different frequency sub-bands, aiding in capturing information from various frequency ranges [68]. Under emotional stimuli, the brain’s responses to different frequencies might vary, potentially relating to emotion regulation and sensory processing. Implementing filter-bank ensemble techniques better captures these frequency-specific variations, thereby enhancing sensitivity to emotion-related changes. Furthermore, integrating the decoding outcomes from the filter bank ensemble through ensemble learning allows us to attain improved results. The covariance matrix-based Riemannian tangent methods offer a robust approach for capturing emotion-related changes, particularly effective due to its ability to model the spatial relationships and interactions between different brain regions, which are crucial in conveying emotional responses [69]. By leveraging adversarial domain adaptation, it is possible to execute adversarial transfer of features between different sensory inputs, enabling the model to better adapt to diverse data distributions. This aids in mitigating the impact of distribution [67,68] disparities among audio, visual, and audio-visual sensory, thereby bolstering the model’s generalization ability in cross-sensory EEG emotion recognition. In addition, by amalgamating explanations from these three facets, our model harnesses the synergy of Filter Bank feature extraction, Riemannian methods, and Adversarial Domain Adaptation to effectively capture and differentiate between emotion-related and sensory-related changes, resulting in a state-of-the-art performance in cross-sensory EEG emotion recognition.

The novelty of this study lies in its novel utilization of Filter Bank Riemannian features in conjunction with adversarial domain adaptation. This combination introduces a perspective methodology to cross-sensory EEG emotion recognition. By amalgamating the explanations above, our model leverages the synergistic interplay of filter bank feature extraction, Riemannian methods, and adversarial domain adaptation to effectively capture and differentiate emotion-related and sensory-related variations. This, in turn, leads to state-of-the-art decoding results in cross-sensory EEG emotion recognition. Simultaneously, ensemble learning with the adapted filter bank Riemannian features helps attenuate the influence of individual-specific frequency band variations potentially caused by cultural, experiential, and background factors, resulting in a reduced standard deviation. In summary, this study significantly advances EEG-based emotion recognition by introducing a comprehensive framework that transcends the limitations of sensory-centric approaches. Through a combination of innovative techniques and various evaluations, the FBADR method not only achieves remarkable accuracy improvements but also ensures reliable performance in complex cross-sensory contexts.

### 4.5. Robustness Verification

Gaussian noise was introduced into the experimental data to further validate the robustness of cross-sensory emotion recognition based on the proposed FBADR. Specifically, with the average power of the original data as $P_{signal}$, the average power of noise as $P_{noise}$, the signal-to-noise ratio (SNR) can be obtained using Equation (9)

(9) $$SNR=10log_{10}\left(\frac{P_{signal}}{P_{noise}}\right)$$

Normally distributed Gaussian noise and the probability density function can be represented by Equation (10):

(10) $$f\left(x\right)=\frac{1}{\sqrt{2\pi }}e^{\left(\frac{x^{2}}{2}\right)}$$

**Figure 13:**
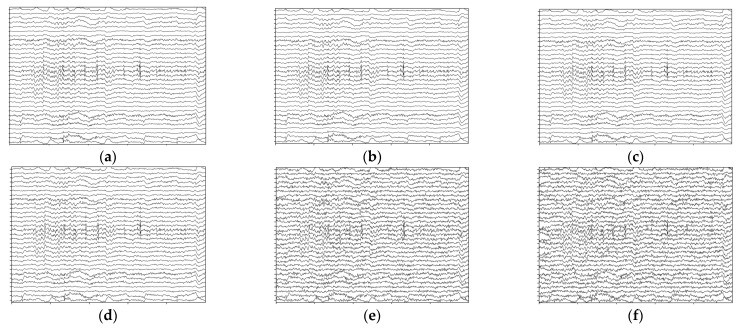
Temporal presentation of the noised signals; data are from the first 5 s of sub 01 audio-happy condition with 1–50 Hz filtered. (**a**) Original data without noise. (**b**) Noised data with SNR=30 dB. (**c**) Noised data with SNR=20 dB. (**d**) Noised data with SNR=10 dB. (**e**) Noised data with SNR=1 dB. (**f**) Noised data with SNR=−0.1 dB.

Coordinated with $P_{signal}$, the $P_{noise}$ varied with SNR=30 dB, 20 dB, 10 dB, 1 dB,−0.1 dB, with the lower signal-to-noise ratio representing a higher power ratio in the noised signals. The temporal presentation of the noised signals is indicated in Figure 13.

**Figure 14:**
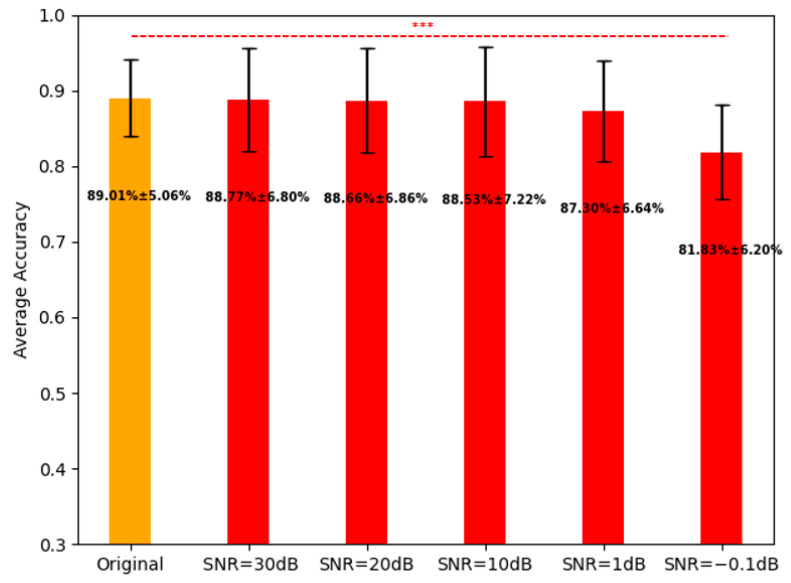
Average classification for the original data and five-group noised data with proposed FBADR, the text average±std of accuracy.

We used the proposed FBADR on the five-group noisy cross-sensory data, and the average classification accuracies for the original data and the five-group noisy cross-sensory data are shown in Figure 14.

Figure 14 indicates that with the noised data of SNR=30 dB, 20 dB, 10 dB, 1 dB, and−0.1 dB, the average accuracy reached 88.77%, 88.66%, 88.53%, 87.30% and 81.83%, respectively. For further statistical analysis a one-way ANOVA was used to determine the statistical difference between the accuracy of the original data and the five-group noised data; p−values were determined from the five pairs as follows: original—SNR:30 dB=0.83, original—SNR:20 dB=0.75, original—SNR:10 dB=0.70, original—SNR:1 dB=0.12, original—$SNR:-0.1\;\mathrm{dB}=2.9\times 10^{-10}$. A p−value of<0.001, denoted as *** in Figure 14, was considered significant. The robustness of the proposed methods could ensure high cross-sensory recognition performance under SNR≥1 dB, making it viable for real-time EEG-aBCI application.

## 5. Conclusions

We introduced a novel framework to address the challenge of cross-sensory EEG emotion recognition in multimodal emotion stimulation as three sensory modalities: audio/visual/audio-visual with two emotion states: pleasure or unpleasure. To accomplish this, we conducted self-designed experiments involving multimodal emotion simulations to acquire cross-sensory emotion EEG data. Our proposed approach—filter bank adversarial domain adaptation Riemann method—leverages Riemannian tangent space methods and filter bank techniques to effectively extract features from cross-sensory emotion data. A key innovation of our study is applying adversarial domain adaptation to mitigate domain differences in cross-sensory situations, enhancing emotion recognition performance. Specifically, we employed adapted features from the source and target domains to train the ensemble SVM classifiers. This integration of adversarial learning and ensemble learning methodologies successfully addressed the challenges associated with cross-sensory EEG emotion recognition, enabling the accurate binary classification of pleasurable and unpleasurable emotions.

We analyzed comparative classification results and demonstrated that our proposed FBADR framework achieved a state-of-the-art performance in cross-sensory emotion recognition, attaining an average accuracy of 89.01%±5.06%. Notably, this level of accuracy was the highest among comparable approaches, accompanied by a low standard deviation. Furthermore, we assessed the robustness of our framework by introducing Gaussian noise, which indicated that the framework was highly resilient to noise interference.

Overall, our study contributes to the field of EEG emotion recognition by offering a comprehensive framework that effectively addresses the challenges in cross-sensory scenarios. Incorporating adversarial domain adaptation and ensemble learning techniques with filter-banked Riemannian features enables accurate emotion classification, while its robustness strengthens its potential for real-world applications.

## Figures and Tables

**Table 1:** Detailed information on the cross-sensory EEG emotion data.

Cross-Sensory EEG Emotion Data Information	
Number of participants	20 (Native Chinese speaker)
Sex	11 males, 9 females
Age	24.7 ± 1.9 years
Number of channels	32 channels
Sampling rate	500 Hz
Experimental stimulus conditions	Audio pleasure/visual pleasure/audio-visual pleasure/audio unpleasure/visual unpleasure/audio-visual unpleasure
Collected EEG data	Each participant: 10 trials with 30 s duration for each condition

**Table 2:** Adaptor structure.

Detailed Parameters in the Adaptor				
Layer	Kernel Size	Output Shape	Activation Function	Batch Normalization
Input	-	961	-	-
Reshape	-	961 × 1	-	-
Conv1D	3	961 × 32	Leaky ReLU	YES
Conv1D	3	961 × 8	Leaky ReLU	YES
Flatten	-	7688	-	-
Dense	-	961	Tanh	-

**Table 3:** Structure of discriminator.

Detailed Parameters in the Discriminator				
Layer	Kernel Size	Output Shape	Activation Function	Batch Normalization
Input	-	961	-	-
Dense	-	32	Leaky ReLU	-
Reshape	2	32 × 1	-	-
Conv1D	2	32 × 32	Leaky ReLU	YES
Conv1D	2	32 × 64	Leaky ReLU	YES
Flatten	-	2048	-	-
Fully connected	-	256	Leaky ReLU	-
Fully connected	-	64	Leaky ReLU	-
Dense	-	1	Sigmoid	-

**Table 4:** Average classification from different SVM kernels of proposed FBADR.

Kernel	Mean Accuracy
Linear	72.49%±9.85%
RBF	81.91%±8.25%
Polynomial	89.01%±5.06%

## Data Availability

Data available on reasonable request due to privacy/ethical restrictions.

## Supplementary Materials

The following supporting information can be downloaded at: https://www.mdpi.com/article/10.3390/brainsci13091326/s1, Table S1: Table of abbreviations, full names and usages.
