# Supplementary material for: Cross-Sensory EEG Emotion Recognition with Filter Bank Riemannian Feature and Adversarial Domain Adaptation
Source: Brain Sci. 2023 Sep 14;13(9):1326. doi: 10.3390/brainsci13091326 (PMC10526196; doi:10.3390/brainsci13091326)
Supplement: Supplementary file 1 [file brainsci-13-01326-s001.zip › brainsci-2565447-supplementary.pdf]

Supplementary material:

Table S1. Table of abbreviations, full names and usages.

Abbreviation	Full name	Usage
EEG	Electroencephalography	Technical term
FBADR	Filter banks adversarial domain adaptation Riemann method	Name of proposed method
SNR	Signal-to-noise ratio	Technical term
a-BCI	Affective brain-computer interface	Technical term
FLD3QN	Frontal Lobe Double Dueling Deep Q Network	Related work introduced in the introduction
TC-Net	Transformer Capsule Network	Related work introduced in the introduction
Bi-LSTM	Bi-directional Long Short-Term Memory	Related work introduced in the introduction
GFIL	Graph-regularized least square regression with feature importance learning	Related work introduced in the introduction
PCC-CNN	Pearson's Correlation Coefficient feature with Convolutional Neural Network	Related work introduced in the introduction and used in baseline comparison.
SVM	Support vector machine	Technical term
ICRM-LSTM	Independent component analysis with Riemannian manifold and long short-term memory networks	Related work introduced in the introduction and used in baseline comparison.
ICA	Independent component analysis	Technical term
daSPDnet	domain-adaptation symmetric positive definite (SPD) matrix network	Related works introduced in the introduction
SPD	Symmetric positive define	Technical term
HMD	Head-mounted displays	Technical term
MED4	Multimodal emotion database	Related work introduced in the introduction
GSR	Galvanic skin response	Technical term
SOGNN	Self-organized graph neural network	Related work introduced in the introduction
CLISA	Contrastive Learning method for Inter-Subject Alignment	Related work introduced in the introduction
FIR	Finite impulse response	Technical term
CM	Covariance matrix	Technical term
TS	Tangent space	Technical term
GANs	Generative adversarial networks	Technical term
LR	Logistic regression	Technical term
RIE	Riemannian method	Relative model used in model self-comparison
ADR	Adversarial domain Riemannian method	Relative model used in model self-comparison

FBR	Filter bank Riemannian method	Relative model used in model self-comparison
TP	True positive	Technical term
TN	True negative	Technical term
FP	False positive	Technical term
FN	False negative	Technical term
ANOVA	Analysis of variance	Technical term
RBF	Radial basis function	Kernel of Support Vector Machine
KNN	K-nearest neighbor	Related work used in baseline comparison.
EEGNET	An end-to-end EEG decoding framework based on neural networks	Related work used in baseline comparison.
PCC-CNN	EEG Emotion recognition framework with Pearson's Correlation Coefficient (PCC) featured images of channel correlation of EEG sub-bands and CNN model to recognize emotion	Related work introduced in the introduction and used in baseline comparison.
DANN	A transfer learning-based EEG emotion recognition framework with adversarial domain adaptation neural networks.	Related work used in the baseline comparison.
WG-DANN	A transfer learning-based EEG emotion recognition framework consists of GANs-like components and a two-step training procedure with pre-training and adversarial training with Wasserstein GAN gradient penalty loss for cross-subject EEG emotion recognition framework.	Related work used in the baseline comparison.
